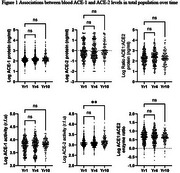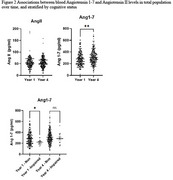# Blood markers of the renin‐angiotensin system and cognitive impairment: The Look AHEAD Study

**DOI:** 10.1002/alz70856_104503

**Published:** 2025-12-26

**Authors:** Sevil Yasar, Andrea Anderson, Kathleen M. Hayden, Mark A. Espeland, Owen T. Carmichael, Jeanne M Clark, Michelle C Carlson, Daniel Asby, Patrick Gavin Kehoe, James Scott Miners

**Affiliations:** ^1^ Johns Hopkins University School of Medicine, Baltimore, MD, USA; ^2^ Wake Forest School of Medicine, Winston‐Salem, NC, USA; ^3^ Wake Forest University School of Medicine, Winston Salem, NC, USA; ^4^ Wake Forest University, Winston‐Salem, NC, USA; ^5^ Pennington Biomedical Research Center, Baton Rouge, LA, USA; ^6^ Rutgers Robert Wood Johnson Medical School, New Brunswick, NJ, USA; ^7^ Johns Hopkins Bloomberg School of Public Health, Baltimore, MD, USA; ^8^ University of Bristol, Medical School, Bristol, United Kingdom; ^9^ University of Bristol, Bristol, United Kingdom; ^10^ University of Bristol, Bristol, Horfield, United Kingdom

## Abstract

**Background:**

The renin‐angiotensin system (RAS) has been proposed as a potential modifier of the development of Alzheimer's disease (AD). However, prospective studies are sparse. We aimed to determine whether plasma ACE‐1, ACE‐2, angiotensin II (ANG‐II), and angiotensin 1‐7 (ANG1‐7) a) change over time, b) differ by cognitive status, c) differ by race.

**Methods:**

We performed a secondary data analysis of the Action for Health in Diabetes (Look AHEAD) study among community‐dwelling, dementia free participants with overweight/obesity and T2DM, aged 45‐76 years at baseline and observed over 12 years. Of 5,145 participants, we included 310 who were not using medications affecting the RAS system, had blood available at follow‐up years 1, 4, and 10, and underwent cognitive adjudication. Plasma ACE and angiotensin were measured using ELISA (R&D systems), and ACE activity was measured using an immunocapture fluorogenic peptide assay. T‐tests were used to test the association between log‐converted ACE‐1 and ACE‐2 at years 1, 4, and 10 and ANGII and ANGII levels at years 1 and 4. We then stratified groups by cognitive status at mean year 11 (cognitively impaired or unimpaired) and race (White and Non‐White [Black and Hispanic)].

**Results:**

ACE‐2 activity between increased significantly between years 1 (1,542  ±  873 rfu and 10 (1,727.05  ±  1,116 rfu) (Figure 1). Similarly, ANG 1‐7 levels increased significantly between years 1 (253.4  ±  80 pg/ml) and 4 (298.4  ±  112 pg/ml) (Figure 2). Cognitively impaired participants (adjudicated between years 8‐12) had significantly lower ANG1‐7 levels (221.0  ±  46 pg/ml) than unimpaired participants (285.8 + 114 pg/ml) in year 1. ANGII levels were significantly lower (61.8  ±  69 pg/ml) in year 1 among Non‐White (mainly Black) compared to White participants (74.8  ±  93 pg/ml).

**Conclusions:**

In this sample of older adults with T2DM and dementia‐free at baseline, we found increased ACE‐2 enzyme over time. We further found lower levels of some of the enzymes in those of non‐white race and those with cognitive impairment. ACE‐2 enzyme is involved in the production of ANG1‐7; thus, the findings of increased ACE‐2 activity associated with increased ACE1‐7 levels are biologically plausible. These findings warrant further evaluation.